# Transcriptome-wide identification and study of cancer-specific splicing events across multiple tumors

**DOI:** 10.18632/oncotarget.3145

**Published:** 2015-02-05

**Authors:** Yihsuan S. Tsai, Daniel Dominguez, Shawn M. Gomez, Zefeng Wang

**Affiliations:** ^1^ Curriculum in Bioinformatics and Computational Biology, University of North Carolina, Chapel Hill, NC 27599, USA; ^2^ Department of Pharmacology, University of North Carolina, Chapel Hill, NC 27599, USA; ^3^ Computer Science, University of North Carolina, Chapel Hill, NC 27599, USA; ^4^ Joint Department of Biomedical Engineering at UNC-Chapel Hill and NC State University, Chapel Hill, NC 27599, USA

**Keywords:** Alternative splicing, Cancer classification, RNA processing, Cell cycle

## Abstract

Dysregulation of alternative splicing (AS) is one of the molecular hallmarks of cancer, with splicing alteration of numerous genes in cancer patients. However, studying splicing mis-regulation in cancer is complicated by the large noise generated from tissue-specific splicing. To obtain a global picture of cancer-specific splicing, we analyzed transcriptome sequencing data from 1149 patients in The Cancer Genome Atlas project, producing a core set of AS events significantly altered across multiple cancer types. These cancer-specific AS events are highly conserved, are more likely to maintain protein reading frame, and mainly function in cell cycle, cell adhesion/migration, and insulin signaling pathways. Furthermore, these events can serve as new molecular biomarkers to distinguish cancer from normal tissues, to separate cancer subtypes, and to predict patient survival. We also found that most genes whose expression is closely associated with cancer-specific splicing are key regulators of the cell cycle. This study uncovers a common set of cancer-specific AS events altered across multiple cancers, providing mechanistic insight into how splicing is mis-regulated in cancers.

## INTRODUCTION

Most human genes undergo alternative splicing (AS) to produce multiple isoforms with different biological properties. This process is tightly controlled across different tissues and developmental stages, and dysregulation of AS is closely associated with various human diseases including cancer [1, 2]. The extensive alteration of AS is considered to be one of the molecular hallmarks of cancer [3], and affects numerous genes that are critical for tumor pathogenesis and progression (e.g. apoptosis, angiogenesis, tumor metastasis) [4]. While most genetic mutations occur at a low frequency in cancer patients (with a few exceptions like TP53), many identified cancer-specific AS events were found in more than half of the tumor samples, suggesting a predominant role of splicing dysregulation in cancer [1, 4]. For example, CD44 is a key mediator of cell-cell and cell-matrix interactions, migration and invasion [5], and different splicing isoforms of CD44 have been linked with tumor evasion and metastasis in many cancers [6–8]. Other well-documented cases include the apoptosis regulator Bcl-x, which can shift its splicing from pro-apoptotic into anti-apoptotic isoforms in cancers [9].

AS is generally regulated by multiple *cis*-acting splicing regulatory elements (SREs) that are specifically bound by *trans*-acting splicing factors to enhance or inhibit the use of nearby splice sites [10, 11]. The same splicing factor may either activate or inhibit splicing by binding to its cognate SREs in different pre-mRNA regions, which is commonly referred to as context dependent activity [12–14]. Various cellular signaling pathways, such as the MEK/ERK or c-Myc pathway [15–17], were found to control the expression level and activity of splicing factors, which in turn determine different splicing patterns in distinct tissues (reviewed in [10, 18]). Many splicing factors are involved in cancer pathogenesis through mediating AS of hundreds of genes [4]. For example, the splicing factor SRSF1 is found to act as a proto-oncogene to promote cell transformation [19], whereas the splicing suppressor RBM4 functions as a tumor suppressor to inhibit tumor progression [20]. These two antagonistic factors control a partially overlapping set of AS events that are involved in cell migration and apoptosis [20].

While the global change of splicing in cancers is being increasingly appreciated, the functional consequences and regulatory mechanisms of cancer-specific AS remain poorly understood. In addition, detailed analyses of cancer-associated splicing were previously focused on single tumor types or specific genes [21, 22], which is often dominated by the noise from tissue specific AS events. Since cancer is a highly heterogeneous disease, the genetic variation between samples has made the identification of cancer-specific splicing isoforms difficult. Recent advance in The Cancer Genome Atlas (TCGA) project has provided tremendous amounts of sequencing data from the transcriptome of thousands of samples in different cancer types [23], making it possible for an unbiased identification and a further analysis of “cancer-specific” splicing events across different cancer types.

In this study, we performed a transcriptome-wide splicing comparison between thousands of tumor samples and paired normal controls to identify a large number of splicing events with altered splicing in cancer. Most of these events were found to change in a single cancer type, and we further identified a core set of cancer-specific AS events across three different cancer types. The genes containing cancer-specific AS events are significantly enriched for functions in cell cycle, cell adhesion/migration, and insulin signaling pathway. Detailed analyses suggested that cancer-specific cassette exons are more conserved among vertebrates and more likely to maintain the protein reading frame. The set of cancer-specific AS events can serve as reliable biomarkers to separate tumor from normal samples and to even distinguish different subtypes of breast cancer. Finally, we found that most genes whose expression is closely associated with cancer-specific splicing are also key regulators of cell cycle, providing a previously unknown link between cell cycle and splicing regulation in cancer cells.

## RESULTS

### Identification of cancer-specific AS events common to multiple cancers

**Figure 1:**
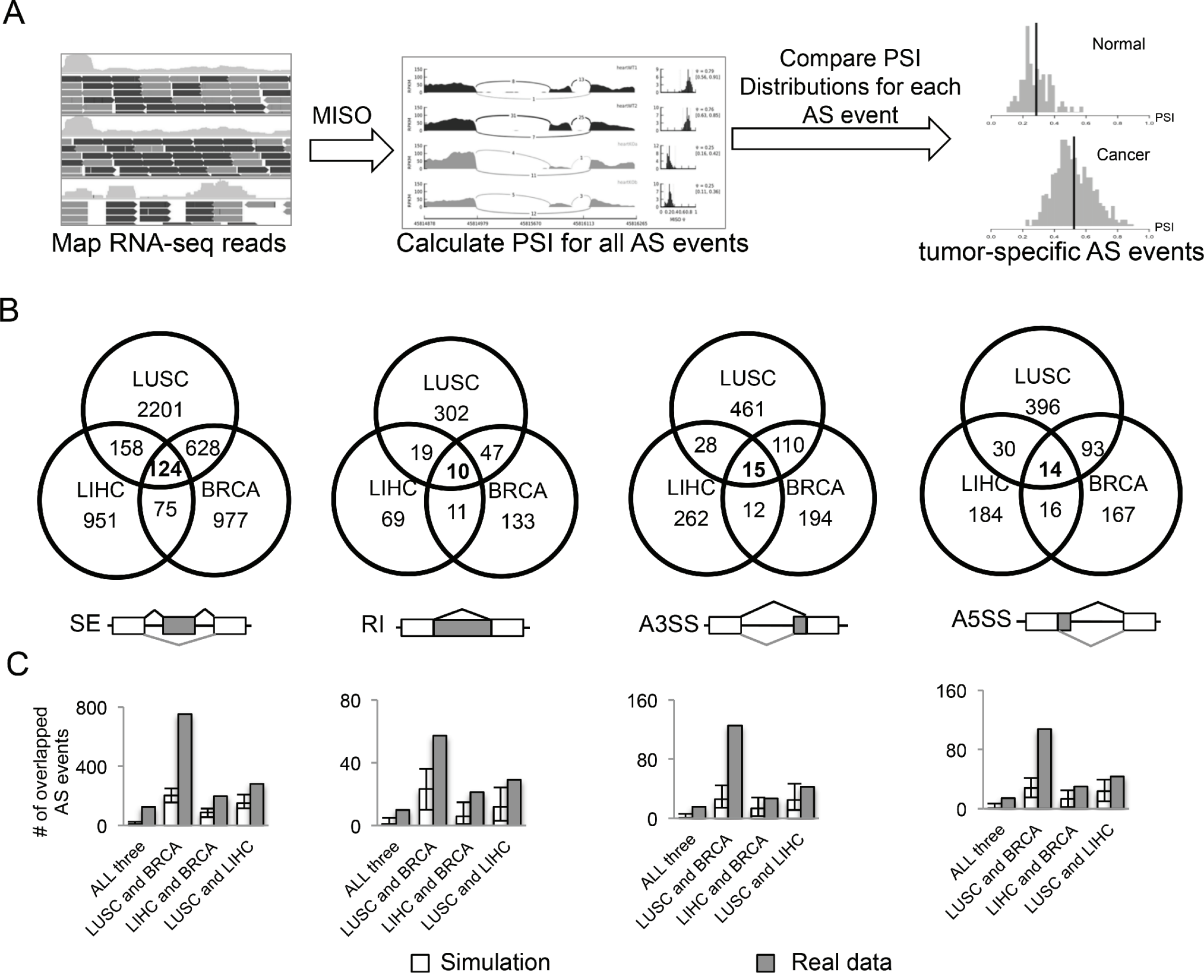
Identification of AS events altered in cancers **(A)** Analysis pipeline to identify AS events that are differentially spliced in cancer *vs*. normal tissues. **(B)** Venn diagram of differentially spliced AS events that are specific to one or multiple types of cancers in four different AS modes (SE, RI, A3SS and A5SS). **(C)** Numbers of AS events overlapped in the simulated dataset (white boxes) and the real dataset (gray boxes). We randomly selected the matched number of AS events from all the detectable AS events for each cancer and then calculated the overlaps. This procedure was repeated 1000 times, and the mean and range of numbers of overlapped events are shown.

It is well known that splicing is controlled in a tissue specific manner with a global change of the splicing landscape between different tissues [24, 25]. Thus identification of AS events altered in cancer vs. normal cells is often complicated by the tissue types used. It remains unclear what portion of AS events are commonly mis-regulated across all cancer types vs. those specific to certain cancers. To identify cancer-specific AS events that are changed within and/or across multiple cancers, we used RNA-seq data collected through TCGA project [23]. The aligned RNA-seq reads were processed through the MISO pipeline to estimate ratios of different splicing isoforms for each annotated splicing event. For each AS event, we calculated the PSI (Percent Spliced In) values between all normal and tumor samples, and identified potential cancer-specific AS events that are significantly altered in cancer vs. normal tissues (Figure 1A).

For a reliable comparison, we selected three types of cancers that have sufficient number of paired normal samples from TCGA, including breast invasive carcinoma (BRCA), lung squamous cell carcinoma (LUSC) and liver hepatocellular carcinoma (LIHC). We focused on four major modes of AS for more detailed analysis: skipped exon (SE), retained intron (RI), alternative 3′ splice site (A3SS) and alternative 5′ splice site (A5SS). In each cancer type, we identified AS events that satisfy the following criteria: (i) the AS event is detected in at least 10 tumor samples and 10 normal samples; (ii) the distributions of PSI values of each event are significantly different between normal and tumor samples (*p* < 0.05 by *t*-test); (iii) the mean difference of PSI values between normal and tumor samples is large than 0.1 (Figure 1B). For each cancer type, we identified several thousand AS events that have significant changes of the splicing isoforms (Table 1). Most of these events are specific to a single cancer type, with lung cancer having more altered AS events compare to breast and liver cancers, probably due to the higher mutation rate in lung cancer [26].

**Table 1:** Summary of cancer dataset

Tumor Type	No. of normal samples	No. of tumor samples	Total mapped Reads	No. of AS events detected	No. of AS events changed in cancer vs. normal
BRCA (breast cancer)	91	727	1.3E + 11	65,152	2,626
LIHC (liver cancer)	36	74	1.6E + 10	70,342	1,978
LUSC (lung cancer)	43	348	6.4E + 10	70,637	4,636

We considered the common events that changed in all three types of cancers as a core set of 163 cancer-specific AS events (Figure 1B and Supplementary Table S1). As a background control, we simulated the overlaps of AS events between different cancer types using 1000 randomly selected datasets with matched size (see methods). In all four AS modes, the overlaps between simulated datasets are significantly smaller than those between real set, especially for the overlaps of all three cancers (Figure 1C). This result suggests that although each cancer type has tissue-specific set of splicing events, there are indeed a significant number of splicing events shared by multiple cancers. Strikingly, 10 of the genes that show cancer-specific AS are also frequently mutated in cancers vs. normal samples [27]. Such overlap is significantly more than the overlap expected by chance (*p* = 10^−6^ by hypergeometric test), indicating that the function of these genes may be altered through either mutation or splicing changes to affect cancer progression. This result also suggests that, in addition to point mutations and copy number variations, the alteration of splicing may serve as another important route to alter gene function in cancer.

Splicing of most introns is regulated in a co-transcriptional fashion [18], thus a change in gene expression may affect AS outcomes of corresponding genes. We examined the genes containing cancer-specific AS events, and found that most of them (68%–90%) do not have significant change of expression levels between normal vs. tumor samples (Supplementary Figure S1A), suggesting that splicing of these events are not biased by their gene expression. In addition, we found that similar numbers of genes have increased vs. decreased PSI values, with exception of RI events that tend to have more retained introns (i.e. PSI increased) in cancers (Supplementary Figure S1B). Intron retention is a relatively less studied mode of AS in mammals and usually changes the coding frame and triggers nonsense mediated mRNA decay (NMD) [28]. Therefore an increase in intron retention may represent an important mechanism for protein inactivation in tumors.

### Consistent change of cancer-specific AS events across tumor types

**Figure 2:**
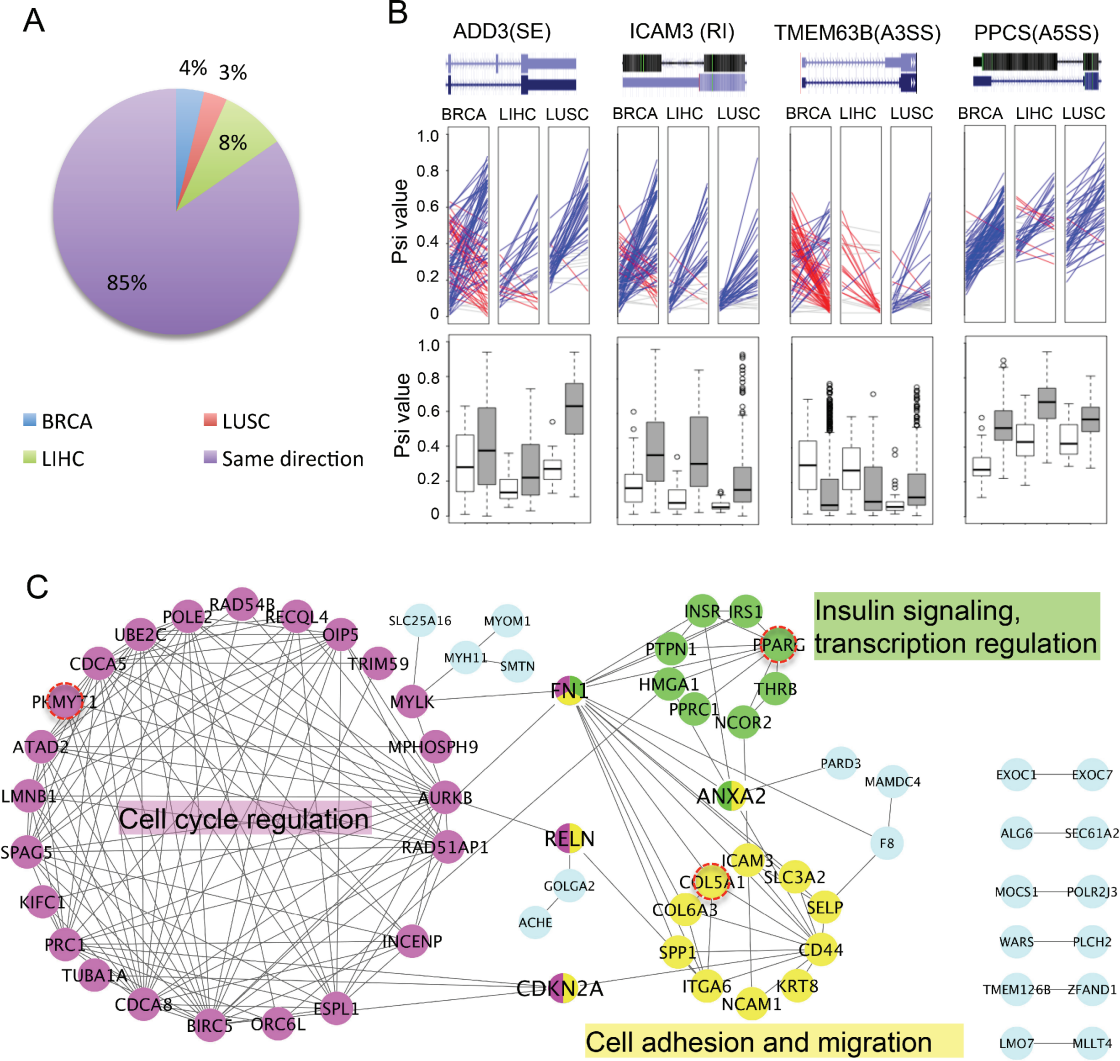
Examples of cancer-specific AS events **(A)** Changes of splicing across different cancer types. For all cancer-specific AS events, the differences of mean PSI values between cancer and normal samples were calculated. We plotted the percent of AS events with PSI changed in same direction among all cancers (purple) or in different directions for one specific type of cancer (BRCA, LUSC or LIHC). **(B)** Change of splicing in selected examples of cancer-specific AS events. The PSI values of paired samples are marked on the left (normal) and right (tumor) panel in each ladder plot with a colored line linking two PSI values. Blue lines represent AS events with increased PSIs in tumor, whereas red lines represent events with decreased PSIs and grey lines represent events with negligible change of PSI (< = 0.05). Box plots in the bottom are comparisons between all normal samples (white boxes) and all tumor samples (grey boxes). **(C)** PPI networks of genes containing cancer-specific AS events. The networks have 3 highly connected clusters defined by MCODE (color coded in pink, yellow and green). The hub proteins interacting with multiple clusters were coded with multiple colors. Three genes that are also frequently mutated in tumors were marked by red circles. The most enriched function/GO-term was labeled next to each cluster.

To determine if the cancer-specific AS events change consistently among different types of cancers, we compared the difference of PSI values between cancer and normal (ΔPSI) across three cancer types for each event (Figure 2A). The majority (i.e. 85%) of these cancer-specific AS events change consistently across different tumor types (i.e., with an increased or decreased PSI values in all three tumors) when comparing tumors to the cognate normal tissue, suggesting that the splicing change in these genes will likely generate similar functional consequences across different tumors. The remaining 15% of AS events, while being altered across all cancers, have different patterns of splicing changes depending on the cancer type.

Representative examples of cancer-specific AS events were arbitrarily selected to illustrate splicing changes between tumor and normal samples. We chose one example from each AS mode and used colored lines to represent the ΔPSI between the paired tumor *vs*. the adjacent normal tissue (Figure 2B, upper panel). We found that there is large heterogeneity of ΔPSI between the paired cancer-normal samples. In some cases of breast cancer, both an increase and decrease in PSI were found among different patients, which might reflect differences between breast cancer subtypes. In addition, we also plotted the distribution of the PSI for the same examples in all normal and tumor samples, and found that the changes of PSI are consistent with those found in paired samples (white and gray boxes, bottom of Figure 2B).

### Biological functions of cancer-specific AS events

We further examined the genes containing newly identified cancer-specific AS events, and found that many of these genes are known to play a key roles in different stages of tumor progression (Supplementary Table S1). Most of these genes are functionally related to each other and form closely connected protein interaction networks (Figure 2C). Based on the MCODE clustering algorithm [29], these genes can be clustered into three groups connected by several hub proteins (Figure 2C and Supplementary Table S2). The largest group contains genes involved in cell cycle regulation (such as AURKB, CDCA5), with genes in the other two groups having functions in mediating cell adhesion/migration (e.g. CD44 and Collagens) and involved in the insulin signaling pathway (e.g. INSR, PPARG). The functional clustering of genes with cancer-specific AS suggests that the regulation of AS in cancer plays important roles in key pathways related to cancer pathogenesis, including cell cycle and cell adhesion/migration. The association of insulin response pathway with splicing regulation in cancer has not been reported before, and its functional implication will be an interesting subject of future studies.

**Figure 3:**
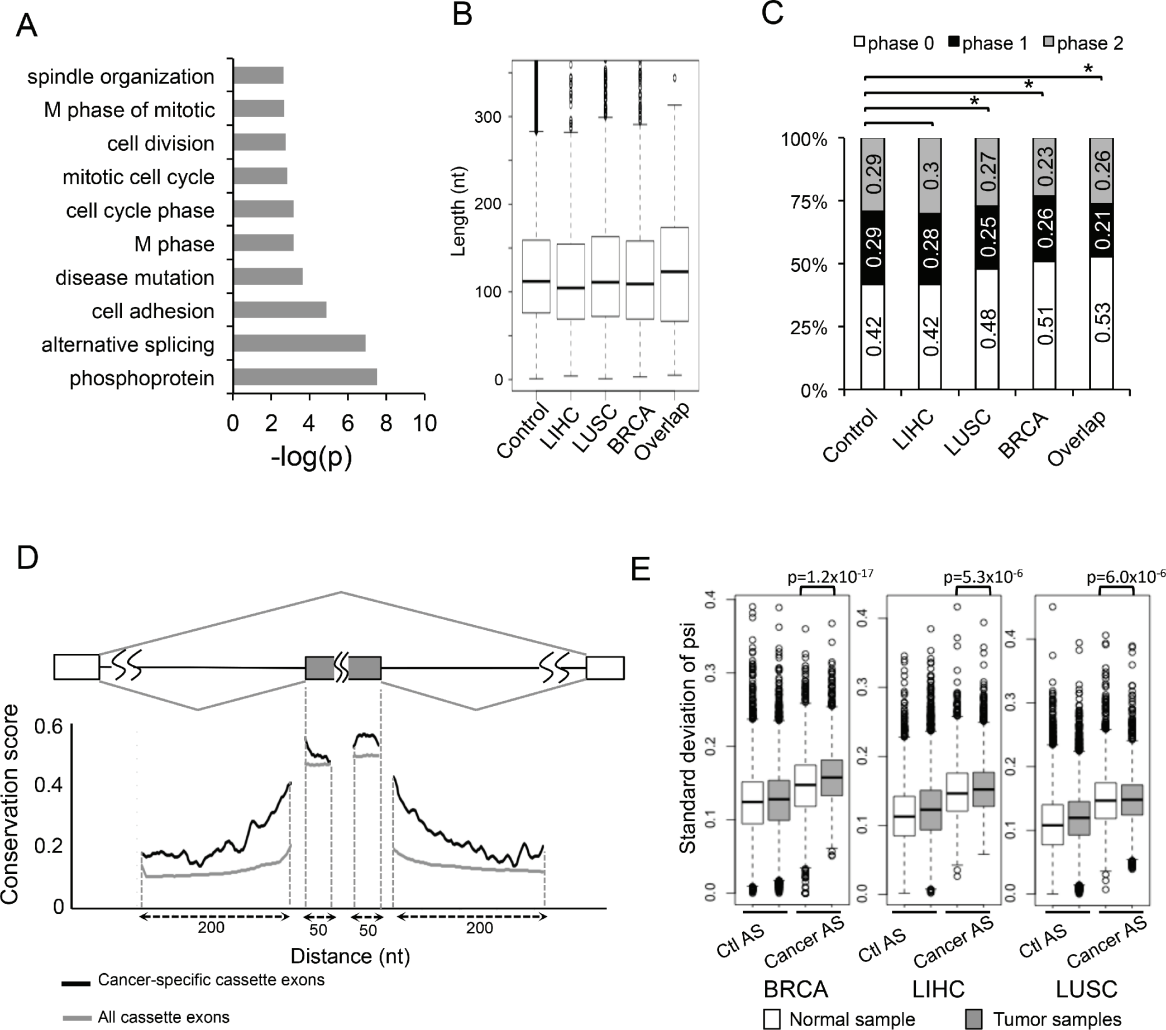
Molecular features of AS events changed in cancers **(A)** The genes containing 163 cancer-specific AS events were analyzed by gene ontology, and the significantly enriched (*p* < 0.005) GO terms are plotted. **(B)** Box plots of the length of alterative exons in the SE events from the MISO database, the SE events that were changed in LIHC, LUSC, BRCA, and the SE events altered in all cancers (from the left to the right). **(C)** Percent of skipped exons in each exon phase. Exons are classified into phase 0, 1, and 2 depending on the reminders when dividing their length by 3. Phase 0 (white boxes): events without frame-shift; Phase1 and 2 (black and gray boxes): events with frame-shift. The order is same as (B). Asterisks indicate significant increase of phase 0 exon (*p* < 0.05 by Fisher's exact test). **(D)** Sequence conservation near the cancer-specific skipped exons and all skipped exons. Black line represents average conservation score from the 124 cancer-specific SE events; grey line represents average conservation score from all the SE events in MISO database (control). **(E)** We compared the distribution of PSI standard deviation between control AS (left) and AS events that change in each cancer (right). We also compared those between normal (white box) and tumor samples (grey box).

To further study the functional consequence of cancer-specific AS events in an unbiased fashion, we performed gene ontology (GO) analysis on genes containing cancer-specific AS events using the DAVID online tool (http://david.abcc.ncifcrf.gov/) [30, 31]. We found that the most enriched functional categories included cell adhesion, cell division, cell cycle and so on. (Figure 3A). We also did GO analysis on each individual cancer type and ranked enriched GO terms by their *p*-value (Supplementary Figure S2A to S2C). The top enriched terms were cytoskeleton proteins and proteins associated with cell adhesion, ATP-binding, cell cycle.

### Sequence characteristics of cancer-specific AS events

The skipped exon is the most common mode of AS among all identified cancer-specific AS events (Figure 1B), providing a sufficient amount of data for detailed sequence analyses. We first measured the length of all skipped exons, and found no obvious difference between the cancer-specific SEs, the SEs that are altered in a single cancer type, and all annotated SEs as control (Figure 3B). We further examined if the lengths of these alternative exons can be divided by three, which is a good indication of how each AS event affects mRNA reading frame. We found that 42% of the alternative exons in control set of SEs are phase 0 exon (i.e. maintain their reading frame), while in the sets of SEs altered in single or multiple types of cancers, a notably increased fraction of exons maintain their reading frame (Figure 3C). In particular, 53% of the SEs shared by all three cancers are phase 0 exons, significantly more than what is expected by chance (*p* = 0.008 by fisher's exact test). Since disruption of reading frame often introduces premature stop codons that lead to NMD, the increased tendency of cancer-specific SEs to maintain reading frame suggests that these events tend to produce proteins with different functions rather than disrupting protein function via changing reading frame.

Alternative splicing is generally regulated by *cis*-acting SREs that function as splicing enhancers or silencers [12]. These SREs usually function in the nearby region of alternative splice sites, and thus the pre-mRNA regions within and adjacent to the alternative exons are more conserved than corresponding regions near the constitutive exons. When further examining the conservation of pre-mRNA regions near 124 cancer-specific SEs shared by three cancer types, we found that these exons tend to be highly conserved across 100 vertebrate species in the adjacent regions. Such sequence conservation is even higher than alternative exons that are spliced in a cancer-independent manner (Figure 3D, comparing black and grey lines), suggesting that cancer-specific alternative exons are under additional evolutionary constraints. This result is consistent with the notion that alternative splicing of cancer-associated genes are tightly controlled in normal cells across different species to mediate critical and highly conserved processes in cell growth.

To further identify putative SREs that control cancer-specific SEs, we examined these highly conserved regulatory regions to measure whether there are enriched sequence motifs that could be potentially recognized by splicing factors (Supplementary Figure S3). Some of these motifs resemble the binding site of known splicing factor. For example, the AC-rich motifs are recognized by hnRNP L and the UG rich motifs resemble hnRNP H/F binding sites [14]. Consistently, the hnRNP H was shown to be up-regulated in certain cancer and control several cancer related splicing events [32, 33].

### Splicing of cancer-specific AS events are highly fluctuated

The ratios between different splicing isoforms of the same gene are tightly regulated to ensure precise control of gene function. In normal cells, splicing is usually controlled in a tissue-specific fashion with certain dominant isoforms in different tissues [24, 25]. However, such dominance of certain tissue-specific isoforms is often absent in cancer cells. In another word, many splicing isoforms are found in the “wrong tissues”, leading to a more dispersed spectrum of AS. However such deregulation of AS in cancer has only been observed in an anecdotal fashion, and a thorough investigation with correct controls is lacking.

To examine potential splicing deregulation in cancers, we directly test: (i) if the splicing of cancer-specific AS events have higher variability than control events, and (ii) if such variability is higher in cancer *vs*. normal samples. We calculated the standard deviation (SD) of PSI value for each AS event, which measures the amount of variation from the average. The SDs of cancer-specific AS events were compared to those of control AS events across both normal and tumor samples in each cancer type. Since the mean value of PSI dramatically affects its SD (Supplementary Figure S4, PSI values near 0 or 1 tend to have smaller SD), we randomly picked control AS events from the MISO database with PSI distribution matched to cancer-specific AS events. The selection of such controls can eliminate potential biases caused by different PSI distribution between cancer-specific AS events vs. all other AS events.

We found that all the cancer-associated AS events have higher PSI variation than controls in all three tissue types (Figure 3E, comparing 2 boxes at the right to the ones at left), suggesting that splicing of these events are indeed highly variable across different samples. In addition, when comparing the cancer-specific AS event in tumor sample with normal samples, those cancer-AS events still tend to have higher variability in tumors than in normal samples (*p*-value: 1.2 × 10^−17^, 5.3 × 10^−6^ and 6.0 × 10^−6^ for BRCA, LIHC and LUSC respectively, Figure 3E). In each cancer type, we also plotted the distribution of PSI in histograms in supplementary figure S5 using both all AS events and the 163 cancer-specific events. This result is consistent with the popular hypothesis that the tissue specificity of AS in normal samples is disrupted in cancers, probably due to extensive changes in the expression levels and/or activities of oncogenic splicing factors.

### Cancer-specific AS events as molecular biomarkers

**Figure 4:**
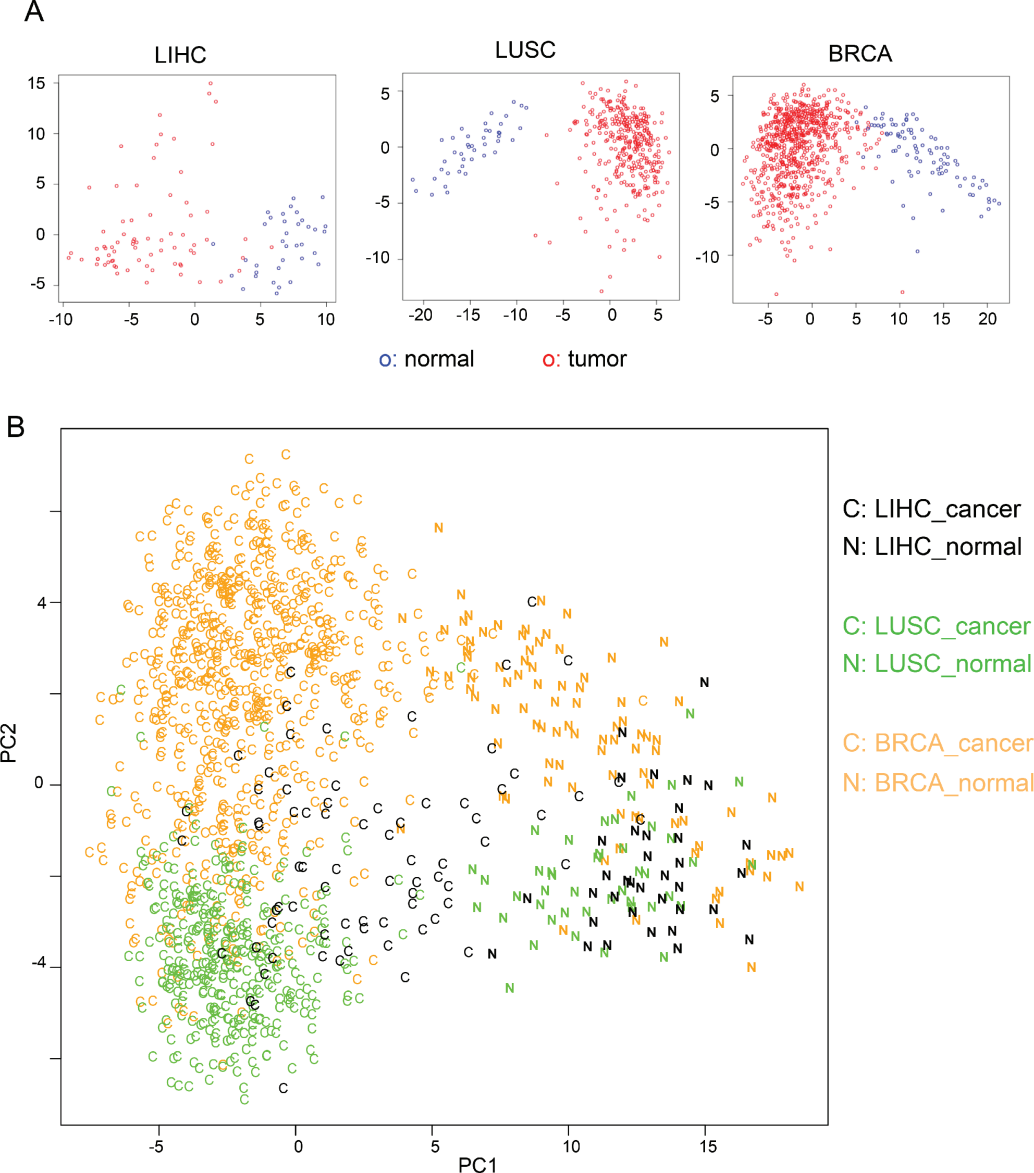
PCA analysis using cancer-specific AS events **(A)** PCA analysis of the 110 liver tissue samples (LIHC: 36 normal and 74 tumor), 391 lung tissue samples (LUSC: 43 normal and 348 tumor) and 818 breast tissue (BRCA: 91 normal and 727 tumor) using the 163 cancer-specific AS events. Tumor samples are in red circles and normal samples are in blue circles. **(B)** PCA analysis of samples from all three tissues using the 163 cancer-specific AS events. Samples were color-coded as its origin tissue. The cancer samples are labeled as “C” and the normal samples are labeled as “N”.

Identification of a core set of cancer-specific AS events makes it possible to use this relatively small dataset as a new molecular biomarker of cancers. To this end, we conducted principal component analysis (PCA) using the 163 cancer-specific AS events. For each tumor or normal sample, we generated a vector with 163 variables using the PSI values of cancer-specific AS events. We constructed a data matrix consist of all tumor and normal samples in each cancer type and further analyzed with PCA. The first two principal components in PCA accounted for 30%, 25% and 24% of the total variance for LIHC, LUSC and BRCA samples respectively (Supplementary Figure S6). The distribution of all samples was plotted using the first two principal components, which show a clear separation between cancer and normal samples (Figure 4A). All analyses showed a reliable separation of samples into two clusters (labeled with red and blue for tumor and normal samples respectively), suggesting that the 163 cancer-specific AS can potentially serve as a reliable biomarker for cancer diagnosis.

In addition, we combined all samples from three types of cancers and analyzed combined data with a similar PCA procedure. Consistent with our analyses of single cancer type, the cancer and normal samples can be reliably separated with the first two principal components (Figure 4B, “C” for cancer and “N” for normal), indicating that cancer-specific AS events are useful molecular biomarkers to separate tumors from mixed samples. In addition, the samples from different tissue types can be roughly separated (Figure 4B, with orange, green and black representing breast, lung and liver respectively). This result suggests that although the 163 AS events are identified based on their altered splicing in multiple cancers, their splicing patterns still partially reflect tissue of origin.

**Figure 5:**
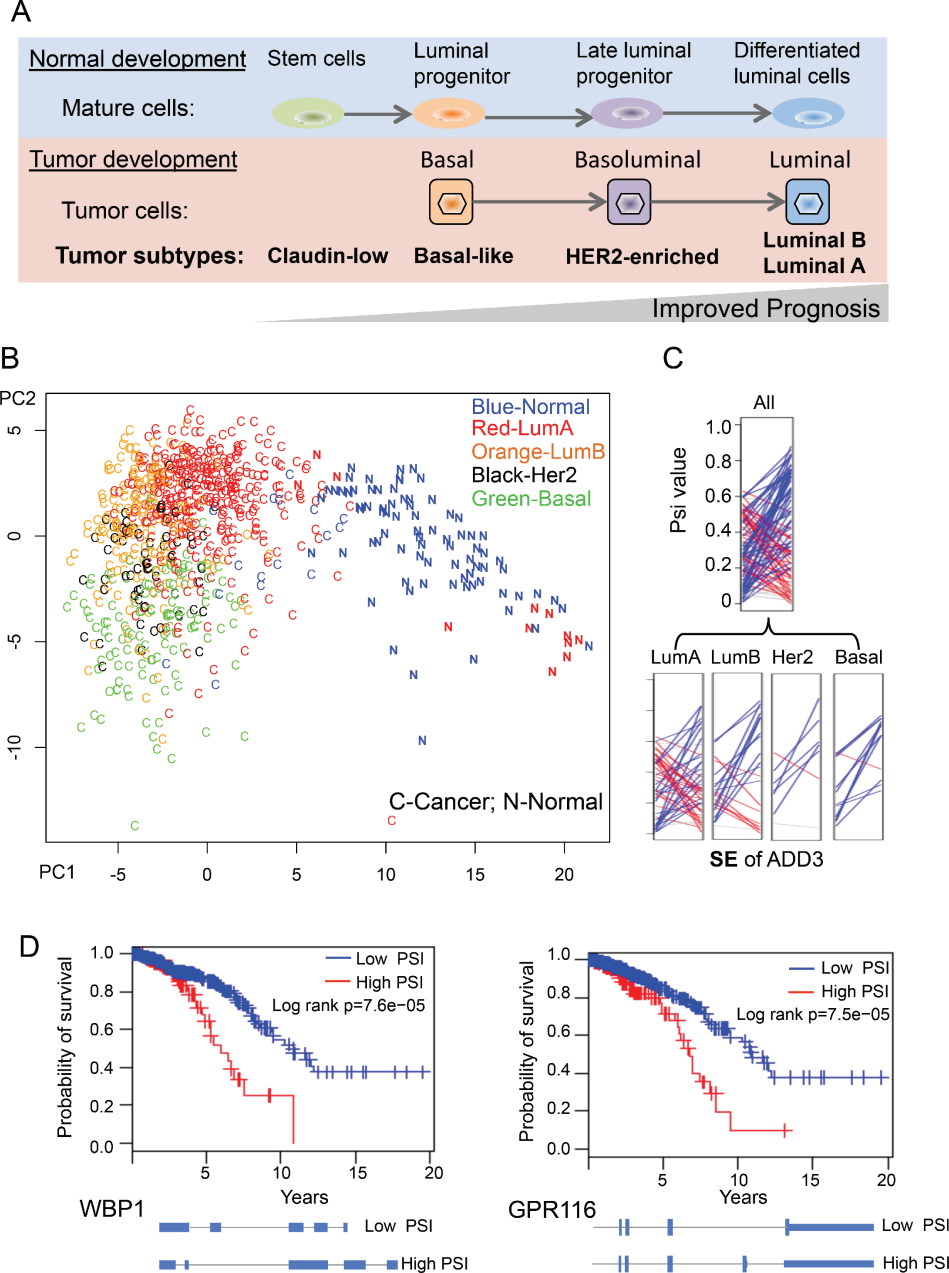
Using cancer-specific AS events to separate breast cancer subtypes **(A)** An overview of four different breast cancer subtypes in tumor developments. **(B)** PCA analysis of the 818 breast tissue using the 163 cancer-specific AS events. Different BRCA samples were labeled according to each subtype as classified by PAM50. **(C)** An example SE event where the different BRCA subtypes have different splicing patterns. The ladder plots were generated as described in figure 2B. **(D)** Two examples of cancer-specific AS events whose PSI value can be used to predict survival of breast cancer patients.

Breast cancer is a well-annotated cancer type in TCGA data and is classified into several subtypes based on histopathological criteria and expression of a core set of genes [34, 35]. The breast cancer cells in different subtypes (Claudin-low, Basal-like, HER2-enriched, Luminial B and Luminal A) resemble cells in different stages of normal mammary development, which is well correlated with tumor progression (Figure 5A) [35]. Since our BRCA dataset has a large number of samples with well-annotated subtype categories, we sought to determine if the cancer-specific AS events can be used to separate cancer subtypes. We conducted a similar PCA procedure using 818 breast samples that were independently classified into normal and four cancer subtypes by PAM50 [34]. By plotting all samples along the first two principal components, we found that different breast cancer subtypes tend to be clustered into different groups (Figure 5B). Certain subtypes of breast cancers, such as basal and luminal types, are particularly well separated. In addition, some normal samples that were misclassified as luminal A cancers by PAM50 were correctly distinguished using cancer-specific AS events, suggesting that the cancer-specific AS events can potentially serve as a cancer biomarker independent of current classification criteria using gene expression data. Although current separation by two PCA components is not very strong with some overlaps between subtypes, this analysis provided a proof-of-concept for splicing-based tumor classification. A more sophisticated statistical approach and analysis is needed to prove this. A representative example of cancer-specific AS events was selected to show that alteration of splicing patterns in the same gene could be different in distinct subtypes of breast cancer (Figure 5C), with the luminal A subtype having the largest variability between patients.

### Ratio of different splicing variants can serve as predictor of cancer survival

Until around 2000, the chance of survival for cancer patients was mainly predicted according to various histologic and clinical characteristics. The advance of microarray technology led to more accurate profiling of gene expression in cancers, allowing prediction of cancer survival by the gene-expression signature of cancer [36, 37].

The extensive splicing mis-regulation and frequent mutations of certain splicing factors in cancer have suggested that some AS events may directly affect tumor biogenesis and progression, however the consequence of splicing mis-regulation on patient survival remains unclear. The identification of cancer-specific AS events across a large number of patients makes it possible to test if splicing misregulation in certain genes can serve as a predictor of cancer prognosis.

We directly test this possibility using TCGA dataset of breast cancer which has largest number of patients. We separated 727 BRCA patients according to the ratio of different splicing isoform for each cancer-specific AS event (i.e. patients with high vs. low PSI values), and examine if such classification is correlated with the overall survival of BRCA patients using Kaplan-Meier analysis. We found that five of cancer-specific AS events indeed can be used as predictor of tumor survival (log rank *p* < 0.05), with two examples shown in figure 5D. The first example, WBP1 (WW domain binding protein 1), is a binding partner of WWOX tumor suppressor that is frequently mutated in breast cancer [38]. We found that increased retention of intron 3 in WBP1 is associated with poor prognosis (Figure 5D). The second example, GPR116, is an adhesion G-protein-coupled receptor that promotes breast cancer metastasis [39]. The inclusion of an alternative exon at end of GPR116 will generate a non-canonical isoform (isoform 2) with a different C-terminal cytoplasmic domain that may change its ability to interact with downstream signaling. We found that the increased production of isoform 2 is associated with poor prognosis. Taken together, our data show that, for the first time, the splicing ratio of some human genes in cancers is associated with cancer survival, suggesting the possibility to use gene splicing as a new molecular signature to predict cancer prognosis.

### Possible regulators of cancer-specific AS

AS is generally controlled by various *trans*-acting splicing factors that specifically recognize *cis*-acting SREs in pre-mRNA. The level and activity of splicing factors usually vary in different cells, leading to the distinct AS patterns in corresponding cell types. Since splicing factors are often controlled by their coexpressed and functionally interacted proteins in different cellular signaling pathways [4], the splicing profile in certain cells may be significantly correlated with the expression of genes that play regulatory roles in AS. Therefore, given a large set of mRNA-seq data across different samples, a global analysis of correlations between AS patterns and expression of all genes may reveal regulatory relationships.

**Figure 6:**
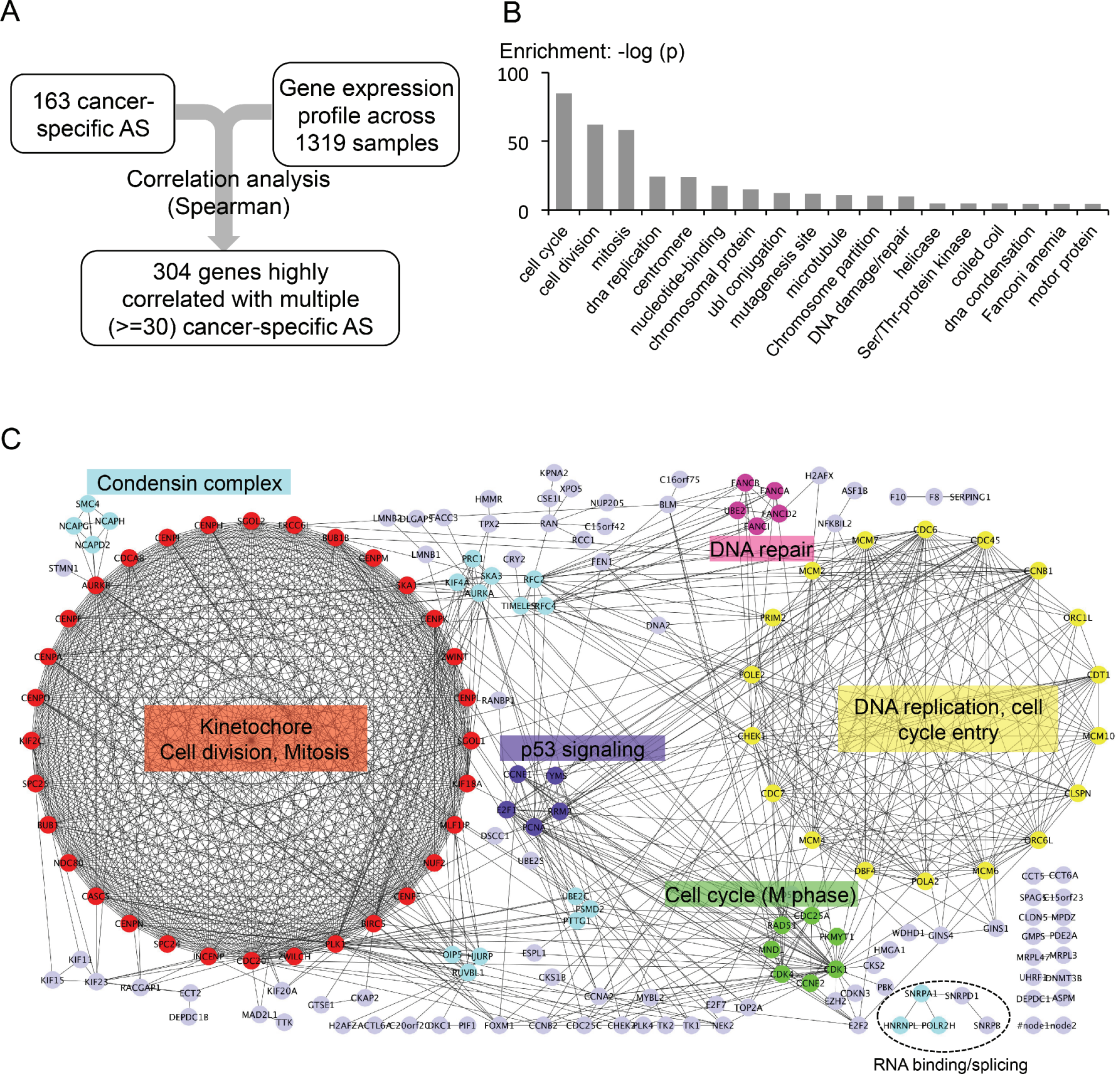
Genes associated with cancer-specific AS events **(A)** Flow chart of identifying possible regulators of cancer-specific AS. **(B)** Gene ontology analysis of the 304 genes that are highly correlated with the 163 tumor AS events. (Spearman rank correlation > = 0.4 across 1319 samples, *p*-value < 0.005, and correlated with more than 30 out of the 163 tumor AS). **(C)** PPI networks of genes that are highly correlated with the 163 cancer-specific AS events. Color-coded proteins are clustered by MCODE. Light purple-colored nodes were proteins that were not clustered into any group by MCODE. The most enriched function/GO-term was labeled next to each cluster. The genes involved in RNA binding and splicing regulation are also indicated at the bottom.

To explore possible regulatory mechanisms of cancer-specific AS, we systematically calculated the correlation between the PSI value of the 163 cancer-specific AS events and the expression of all detectable genes (Figure 6A). We found that the set of cancer-specific AS events are indeed significantly correlated with expression of many genes, among which are 304 genes highly correlated with more than 30 cancer-specific AS events. This set of genes are either positively or negatively correlated with the PSI values of many cancer-specific AS events, and thus may reflect potential regulatory pathways for the associated AS events.

We further conducted GO analysis on genes significantly associated with cancer-specific AS events, and found that the vast majority of them function in multiple pathways related to cell cycle regulation (Figure 6B). However, the functional category of RNA-binding is not significantly enriched in the set of genes associated with cancer-specific AS. We were a little surprised by the small number of correlated splicing factors. However, splicing factor activities can also be regulated in the level of protein modification, and indeed we found that the phosphorylation of several splicing factors is cell cycle dependent (Dominguez at al, unpublished data). This result suggests that the activity of splicing factors may be controlled in a cell cycle dependent manner (e.g., through protein phosphorylation), and thus cell cycle proteins can indirectly affect splicing in tumor cells.

In addition, we analyzed protein-protein interaction among the genes correlated with cancer-specific AS using the STRING database. We found that 173 out of the 304 genes are highly connected with each other, and surprisingly the two largest clusters in the interaction network consist predominantly of genes that mediate the two major cell cycle checkpoints (i.e. checkpoint for G1-S and G2-M transition, Figure 6C). The genes in the largest cluster remarkably form a complete graph with 32 nodes, of which each connects with all others to function in kinetochore formation, cell division and mitosis (red cluster in Figure 6C, also see Supplementary Table S3). The second biggest group has 18 genes that are mostly involved in DNA replication. Even the smaller clusters have cell cycle related functions such as P53 signaling, M phase, DNA repair, and condensin complex. Such a high degree of correlation between cell cycle regulation and cancer-specific splicing has not been previously reported but has profound implication in how AS is controlled in multiple cancers.

## DISCUSSION

Here we performed a systematic identification and analysis of cancer-specific AS events using thousands of patient samples from TCGA data. To increase the statistical power of our analyses, we selected three types of cancers that have a relatively large number of paired normal controls. These tissue types are sufficiently different to enable us to filter out tissue-specific splicing, as most identified AS events are altered in only a single type of cancer (Figure 1B). The AS events significantly altered in all three cancer types include many genes whose splicing was known to play critical roles in cancer development, such as the CD44 [8], NUMB [40, 41], and FN1 [42]. These genes probably represent a core set of cancer-specific AS events that affect key pathways in cancer progression.

On the other hand, several AS events that have well-known roles in cancer development were not identified by our procedure, probably due to the high stringency used in our filters. For example, our set of cancer-specific AS does not include Bcl-x, whose splicing is known to control cell apoptosis in multiple tumors [9] and can be used as a potential therapeutic target [43, 44]. However the ΔPSI of Bcl-x is not large enough to pass the thresholds in our pipeline, and we expect that additional cancer-specific AS events can be identified when the criteria are relaxed. We also require the AS events to be detected in ~30% of normal liver samples (10 out of 36, see table 1), which may cause some uncommon events to be omitted in our pipeline.

Although most cancer-specific AS events are involved in cellular pathways critical to cell growth and migration, they may not directly drive the initial stages of tumorigenesis, as we could not detect obvious mutations near the splice sites of these alternatively spliced exons. Instead we speculate that they are more likely to be a result of mis-regulated splicing factors that potentially change splicing of many pre-mRNA targets. Consistently, several splicing factors were found to be mutated or significantly changed in expression between cancers and normal tissue, including SRSF1, QK1, RBM4, RBM5/6/10, and hnRNP A2 [19, 20, 40, 41, 45]. These results imply that cancer-specific AS events will be more useful as cancer biomarkers, whereas the splicing factors may better serve as potential therapeutic targets to restore mis-regulated splicing in cancer.

To study potential regulatory mechanisms for the cancer-specific AS events, we used an association study to identify genes whose expression is correlated with these events across thousands of tumor and normal samples. This large dataset size enables a statistically reliable identification of genes that directly or indirectly regulate AS. Such analyses only identified a small number of putative splicing factors including hnRNP L and snRPA1 (Figure 6C, marked at the bottom). We speculate that this is due to the large heterogeneity among tumor samples, as the known cancer-related splicing factors are found to be altered in only a subset of tumor samples. Remarkably, the majority of genes whose expression is associated with cancer-specific AS events are those involved in cell cycle regulation, revealing an unknown link between cell cycle and splicing regulation. An unbiased clustering of these associated genes recapitulated two major cell cycle checkpoints (i.e., G1 to S and G2 to M transition) and several main control pathways for cell cycle progression (e.g., DNA repair and P53 signaling). Although the reason of such high correlation is not clear, there are several interesting implications and predictions. For example, this result may suggest that genes controlling cell cycle progression also play a central regulatory role in pre-mRNA splicing and processing. Since cancer cells undergo fast growth and division compared to normal cells, there may be an increasing pressure for cancer cells to transcribe and splice certain genes at a high rate in some cell cycle stages. Because most introns are spliced co-transcriptionally, the increased transcription rate may directly affect AS of a certain set of genes in cancer. There may also be epigenetic factors that bridge the regulation of cell cycle with alternative splicing. A careful examination of this link requires integration of the changes in various epigenetic markers and transcription factors with splicing alteration, which will be an important direction for future investigation. Another interesting implication is that AS may be temporally regulated during the cell cycle. Although periodic gene transcription during cell cycle is well documented [46], there are limited reports on temporal regulation of splicing at different cell cycle stages. Our result implies that such a regulation mode is likely to exist and may even be a major mechanism responsible for cancer-specific AS.

In summary, this study generated a common set of cancer-specific AS events across different cancer types, which can be used as novel cancer biomarkers. We provided a detailed picture of unique features for these AS events and mechanistic insights on how splicing is mis-regulated in cancer. Because dysregulation of splicing in cancer can often serve as a cancer progression indicator, the identification of a core set of cancer-specific AS events will likely help early cancer detection and thus improve the chance of cure. Finally, this relative small set of AS events will facilitate direct discovery of key regulators that are responsible for splicing dysregulation in cancers and thus can potentially be used as new therapeutic targets.

## MATERIALS AND METHODS

### Data acquisition and sequence processing

Pair-ended RNA-seq data were acquired from the TCGA consortium, with each sample having an average of > 150 million reads. The reads were aligned to the human genome version hg19 with MapSplice V2.0 [47], and the gene expression values were estimated using the RSEM pipeline [48] and normalized to the upper quartile of all expressed genes [49].

To analyze AS events on a genomic scale, we used the MISO [50] event-centric pipeline with the hg19 v2.0 annotation to calculate the inclusion ratio of all annotated AS isoforms. To qualify as a valid AS events, we require that both isoforms are detectable in at least 10 normal samples and 10 tumor samples for each cancer type. See Supplementary Methods for details.

### Determination of AS events shared between cancer types

To examine the statistical significance for the number of AS events that are in common between different cancers, 1000 simulated datasets were generated by randomly selecting a control set of AS events with matched size in each cancer type (number of AS events for lung, liver and breast cancer respectively: SE: 3111, 1308, 1804; RI: 378, 109, 201; A3SS: 614, 317, 331; A5SS: 533, 244, 290). We then computed the number of events that were common across multiple cancer types. In each AS mode (SE, RI, A3SS and A5SS), we generated 1000 simulated datasets and calculate the mean overlaps between different cancers, which were then compared to the overlaps of real data using rank test.

### Motif enrichment analysis

The enriched RNA motifs near the splice sites of cancer-specific AS events were calculated by counting all 5-nt “words” near the alternative exons and computing the Z-score as described earlier [51]. All 5-mers with Z-score larger than 2.5 were then clustered by sequence similarity and multiply aligned by using CLUSTALW to identify candidate motifs. See Supplementary Methods for details.

### Principal component analysis (PCA)

PSI values of the 163 cancer-specific AS events were used to form the data vector for PCA. For each cancer type, the PSI vectors across all normal and tumor samples were then combined and used as the input data matrix to perform PCA. The distribution of normal and cancer samples across the first two components were plotted. See Supplementary Methods for details.

### Correlation between gene expression and AS

Correlations between genes and AS events were calculated using two matrices. The first matrix consists of the PSI values of 163 cancer-specific AS events across 1319 cancer and normal samples. Another matrix contains the expression level of every gene across 1319 samples. We computed the spearman rank correlation, ρ (rho), between every two vectors from the two matrices. Each pair with |ρ| > = 0.4 and *p* < = 0.005 was considered as a highly correlated event-gene pair. See Supplementary Methods for details.

## SUPPLEMENTARY METHODS




