# Supplementary material for: Transcriptome-wide identification and study of cancer-specific splicing events across multiple tumors
Source: Oncotarget. 2015 Feb 5;6(9):6825–39. doi: 10.18632/oncotarget.3145 (PMC4466652; doi:10.18632/oncotarget.3145)
Supplement: Supplementary file 1 [file oncotarget-06-6825-s001.pdf]

SUPPLEMENTARY METHODS

Data acquisition and sequence processing

RNA-seq data were acquired from the TCGA consortium, with all reads being pair-ended (length: 50, 48, and 48 for breast, lung, and liver cancer respectively). Each sample has an average of > 150 million reads. The reads were aligned to the human genome version hg19 with MapSplice V2.0 [1], and the gene expression values were estimated using the RSEM pipeline [2] and normalized to the upper quartile of all expressed genes [3].

To analyze AS events on a genomic scale, we used the MISO event-centric pipeline with the hg19 v2.0 annotation to calculate the inclusion ratio of all annotated AS isoforms (http://genes.mit.edu/burgelab/miso/annotations/ver2/miso_annotations_hg19_v2.zip). Further analyses were carried out for four major modes of AS: skipped exon (SE), retained intron (RI), alternative 3' splice site (A3SS) and alternative 5' splice site (A5SS). Based on the coverage of different splicing isoforms, each AS event was assigned with a PSI (Percent Spliced In) value ranging from 0 to 1. To qualify as a valid AS events, we require that both isoforms are detectable in at least 10 normal samples and 10 tumor samples for each cancer type.

Analyses of protein-protein-interaction among cancer-specific AS events

The genes containing cancer-specific AS events (or genes whose expression is associated with cancer-specific AS events) were obtained and submitted to the STRING database [4, 5] (http://string-db.org/) for protein-protein interactions (PPI) analysis. We used the combined score of 0.4 as a cutoff and included five white nodes for network continuity. We used Cytoscape [6] to visualize the PPI network and the MCODE algorithm [7] to identify highly connected clusters within the network. See supplementary Table 2 and 3 for detailed parameters.

Calculation of evolutionary score

Sequence evolutionary score was downloaded from UCSC phastCons100 (http://hgdownload.cse.ucsc.edu/goldenPath/hg19/phastCons100way/) [8]. Based on multiple sequence alignments of 100 vertebrate species, each nucleotide was given an evolutionary conservation score ranging from 0 to 1. Highly conserved regions are assigned with a higher score. PhastCons estimates the probability that each nucleotide belongs to a conserved element based on multiple alignments using a hidden Markov model. For each SE event, we extracted sequences from different regions near the alternative exon to calculate average conservation score in a sliding window of 8 nt across all cancer-specific SE events and control events.

Motif enrichment analysis

To analyze the enriched sequence motifs near the splice sites of the 163 cancerspecific AS events, we first obtained nucleotide sequences from three splicing regulatory regions: upstream intron (300 nt), exon and downstream intron (300 nt) as shown in Figure S3. When obtaining the sequences, we excluded the first 25 nucleotides right upstream of the skipped exon, the first 10 nucleotides right downstream of the skipped exon and the first and the last two nucleotides within the exon. We then calculated the frequency and Z-score of each 5-mer sequence from all 163 sequences in three regulatory regions using methods described in [9]. All 5-mer sequences with Z-score larger than 2.5 were then clustered by sequence similarity and multiply aligned by using CLUSTALW to identify candidate motifs. At a cutoff dissimilarity score of 2.65, 2.7 and 2.7, we obtained 5, 7 and 5 clusters of at least four sequences in each cluster for upstream intron, exon and downstream intron respectively. Finally, we plotted the consensus sequence for each cluster for all three regulatory regions (Fig. S3).

Principal component analysis (PCA)

PCA is a data analysis technique commonly applied for dimension reduction, exploratory analysis and feature selection. PSI values of the 163 cancer-specific AS events were used to form the data vector for PCA. For each cancer type, the PSI vectors across all normal and tumor samples were then combined and used as the input data matrix to perform PCA using the prcomp() function in R. We also conducted PCA by combining the PSI values across all samples from three cancer types. The distribution of normal and cancer samples across the first two components were plotted.

Survival analysis for breast cancer patient

We obtained the overall survival data of breast cancer patients from the UCSC Cancer Browser (727 patients). If a patient deceased (event happened), the "days_to_death" was used as the time variable; if a patient is still living, the time variable is the maximum of "days_to_last_known_alive" and "days_to_last_followup". The patient samples were split into two groups according

to the top or bottom quantile of PSI values for each of the 163 cancer-specific events. The resulted two patient groups are compared for their probability of survival using a Kaplan-Meier survival plot and the logrank P values are calculated. This process was repeated for every cancerspecific event.

Correlation between gene expression and AS

Correlations between genes and AS events were calculated using two matrices. One matrix consists of the PSI values of 163 cancer-specific AS events across 1319 cancer and normal samples. Another matrix contains the expression level of every gene across 1319 samples. We computed the spearman rank correlation, ρ (rho), between every two vectors from the two matrices using cor.test() in R. Each pair with $|\rho| >= 0.4$ and $p <= 0.005$ was considered as a highly correlated event-gene pair. We considered genes that are highly correlated with more than 30 cancer-specific AS events as potential regulators through a direct or indirect regulation. We then used STRING database [4, 5] (http://string-db.org/) to extract PPIs between these potential regulators (304 genes), and Cytoscape and MCODE to visualize and cluster the interaction networks.

REFERENCE

1. Wang K, Singh D, Zeng Z, Coleman SJ, Huang Y, Savich GL, He X, Mieczkowski P, Grimm SA, Perou CM, MacLeod JN, Chiang DY, Prins JF, Liu J. MapSplice: accurate mapping of RNA-seq reads for splice junction discovery. Nucleic acids research. 2010; 38:e178.
2. Li B, Dewey CN. RSEM: accurate transcript quantification from RNA-Seq data with or without a reference genome. BMC Bioinformatics. 2011; 12:323.
3. Bullard JH, Purdom E, Hansen KD, Dudoit S. Evaluation of statistical methods for normalization and differential expression in mRNA-Seq experiments. BMC Bioinformatics. 2010; 11:94.
4. Snel B, Lehmann G, Bork P, Huynen MA. STRING: a web-server to retrieve and display the repeatedly occurring neighbourhood of a gene. Nucleic acids research. 2000; 28:3442–3444.
5. Franceschini A, Szklarczyk D, Frankild S, Kuhn M, Simonovic M, Roth A, Lin J, Minguez P, Bork P, von Mering C, Jensen LJ. STRING v9.1: protein-protein interaction networks, with increased coverage and integration. Nucleic acids research. 2013; 41:D808–815.
6. Shannon P, Markiel A, Ozier O, Baliga NS, Wang JT, Ramage D, Amin N, Schwikowski B, Ideker T. Cytoscape: a software environment for integrated models of biomolecular interaction networks. Genome research. 2003; 13:2498–2504.
7. Bader GD, Hogue CW. An automated method for finding molecular complexes in large protein interaction networks. BMC Bioinformatics. 2003; 4:2.
8. Siepel A, Bejerano G, Pedersen JS, Hinrichs AS, Hou M, Rosenbloom K, Clawson H, Spieth J, Hillier LW, Richards S, Weinstock GM, Wilson RK, Gibbs RA, Kent WJ, Miller W, Haussler D. Evolutionarily conserved elements in vertebrate, insect, worm, and yeast genomes. Genome research. 2005; 15:1034–1050.
9. Fairbrother WG, Yeh RF, Sharp PA, Burge CB. Predictive identification of exonic splicing enhancers in human genes. Science. 2002; 297:1007–1013.

SUPPLEMENTARY FIGURES AND TABLES

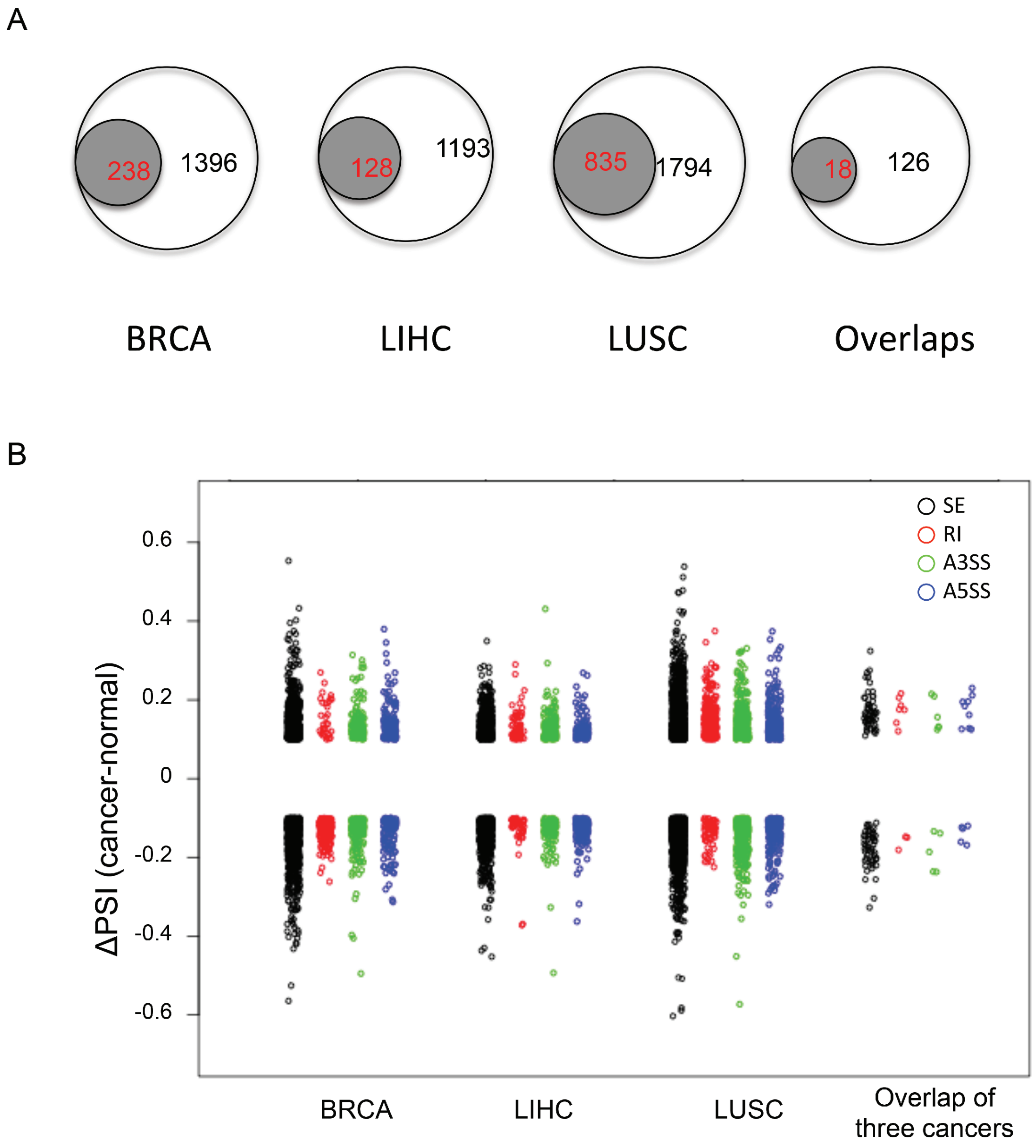

Supplementary Figure S1: The percentage of genes change in both expression level and splicing and the splicing isoform change in four AS modes. (A) For the genes containing cancer-specific AS (white circle) in each cancer type, a small fraction also showed significant changes in expression level between tumor and normal samples (grey circle). We used the following threshold for expression changes: the expression levels of each gene have to change by at least two fold between cancers *vs* normal with *p*-value <= 0.005. **(B)** The change of PSI values between paired tumor and normal samples were plotted in each cancer type and in all three cancers separately. ΔPSI was calculated as the PSI value in cancer sample minus the PSI value in paired normal control. Each dot represents a paired of cancer and normal sample.

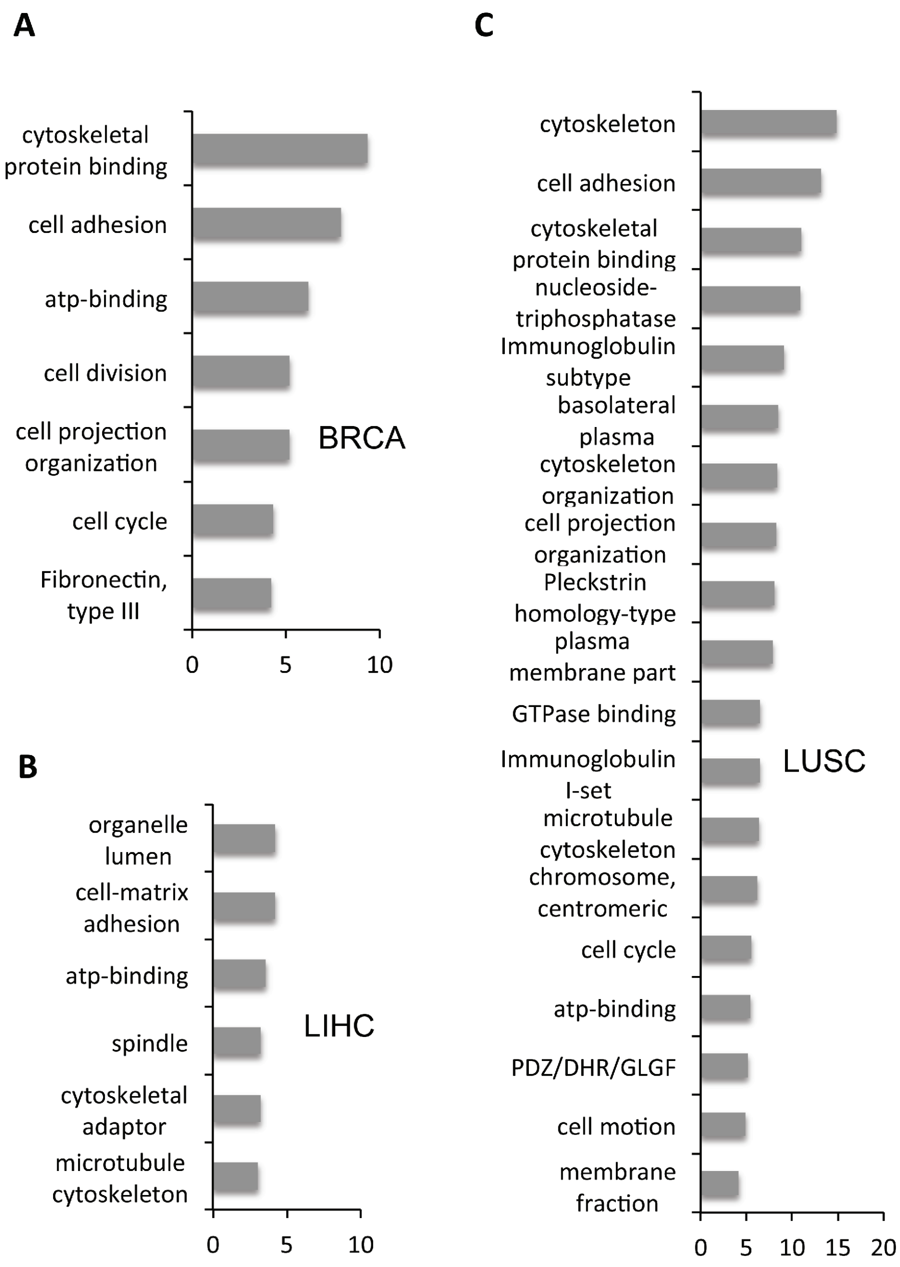

Supplementary Figure S2: Gene ontology analysis of AS events altered in three cancer types: BRCA (A), LIHC (B) and LUSC (C). We obtained the list of genes containing AS events that change significantly in their PSI values between tumor and normal samples in breast, liver and lung cancer datasets, and listed the highly enriched GO terms with pvalue less than 0.005 using DAVID gene ontology tool. The x-axis is the −log (P) of the enriched GO term.

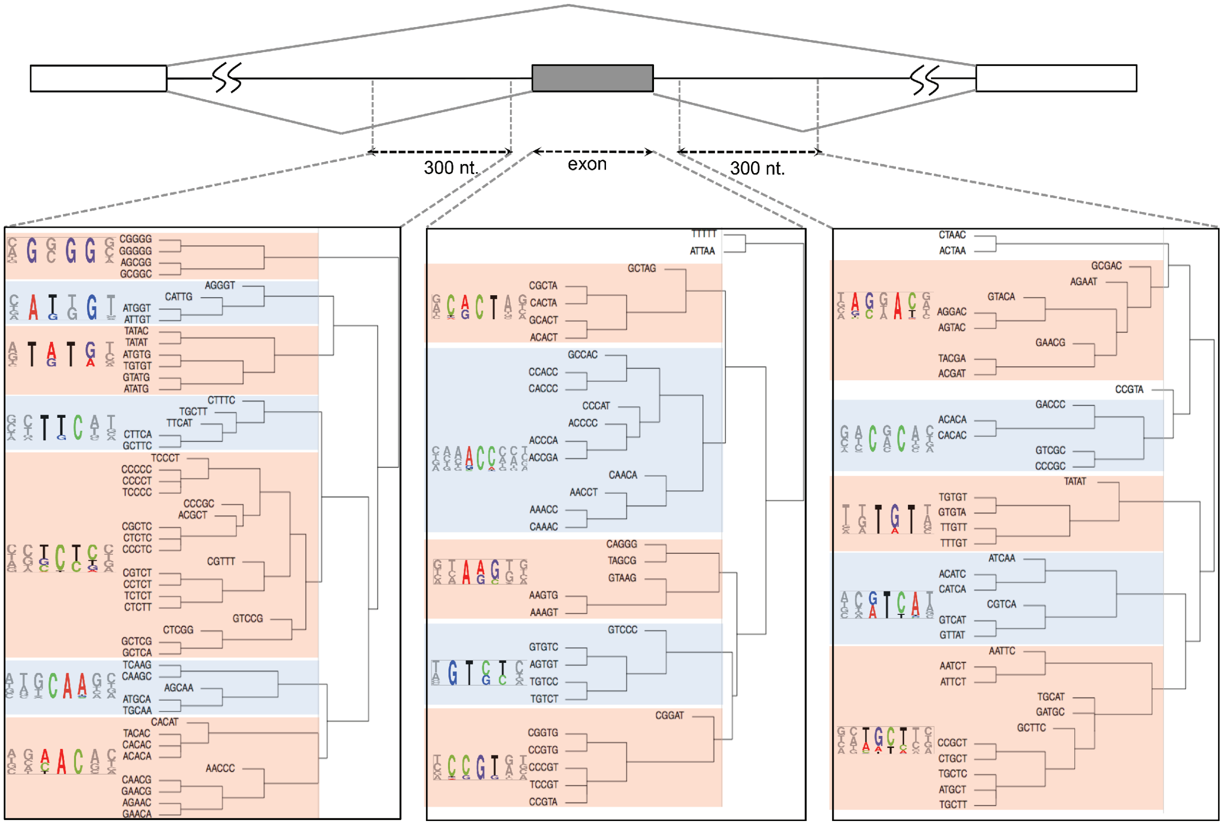

Supplementary Figure S3: Enriched motifs near cancer-specific skipped exons. The pentamers significantly enriched in each pre-mRNA region were identified and clustered into different groups according sequence similarity. The consensus motif in each group was represented with pictogram. Upstream intron: 300 nucleotides upstream of the skipped exon. The enriched Exon region: the whole exon sequences were used except the first and the last two nucleotides. Downstream intron: 300 nucleotides downstream of the skipped exon. The regions overlapping with splice sites (the first 10 nt and the last 25 nt of intorns) were excluded to avoid strong splicing signals.

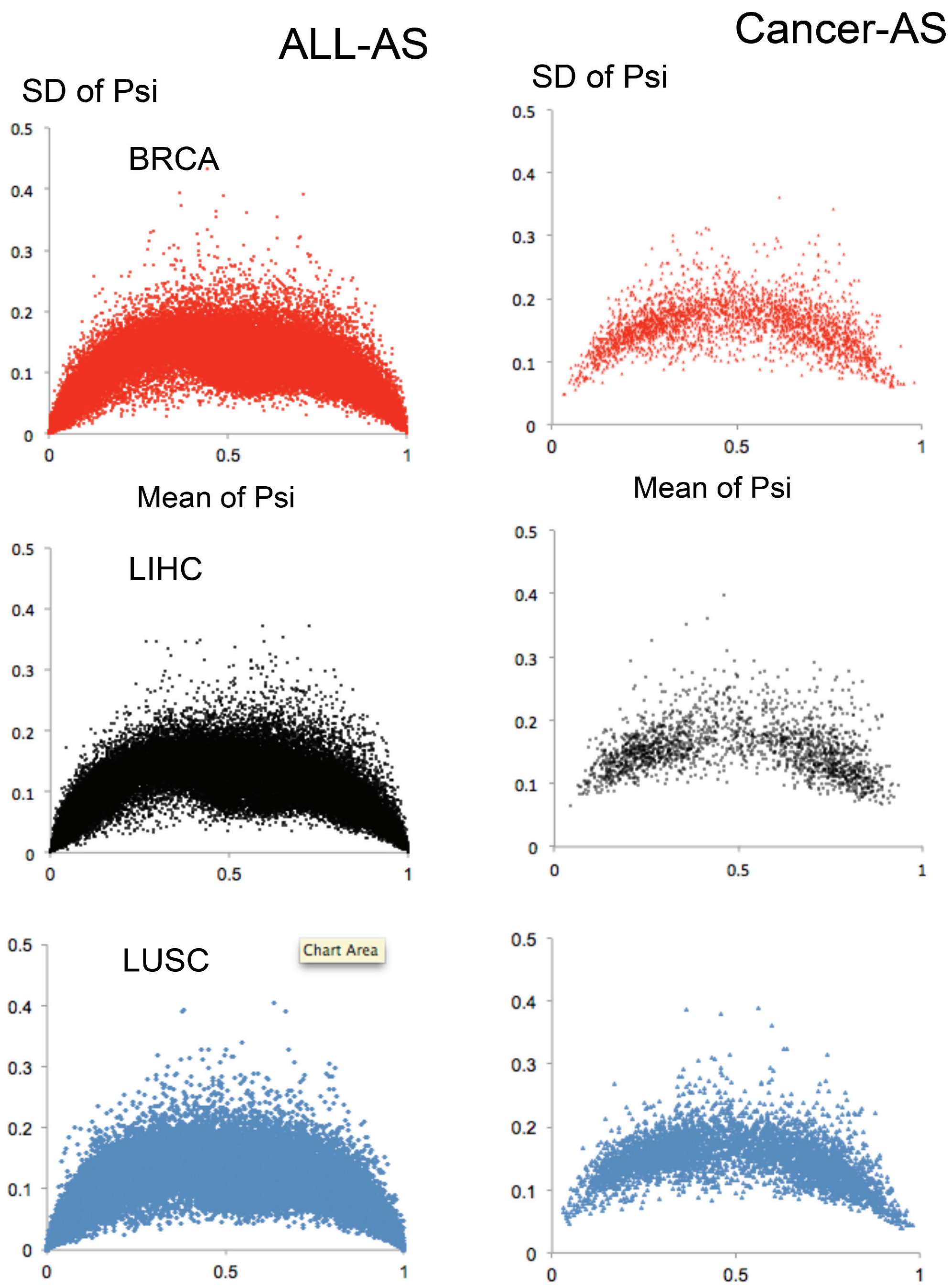

Supplementary Figure S4: Scatter plots of the standard deviation of PSI vs. mean of PSI. For each AS event, the PSI values and the standard deviation of PSI were plotted among all samples in breast, liver and lung cancer datasets. The distribution of all AS events (left) were compared to the AS events that significantly change between tumor and normal (right), and the control set was selected from all AS events with matched distribution of PSI values.

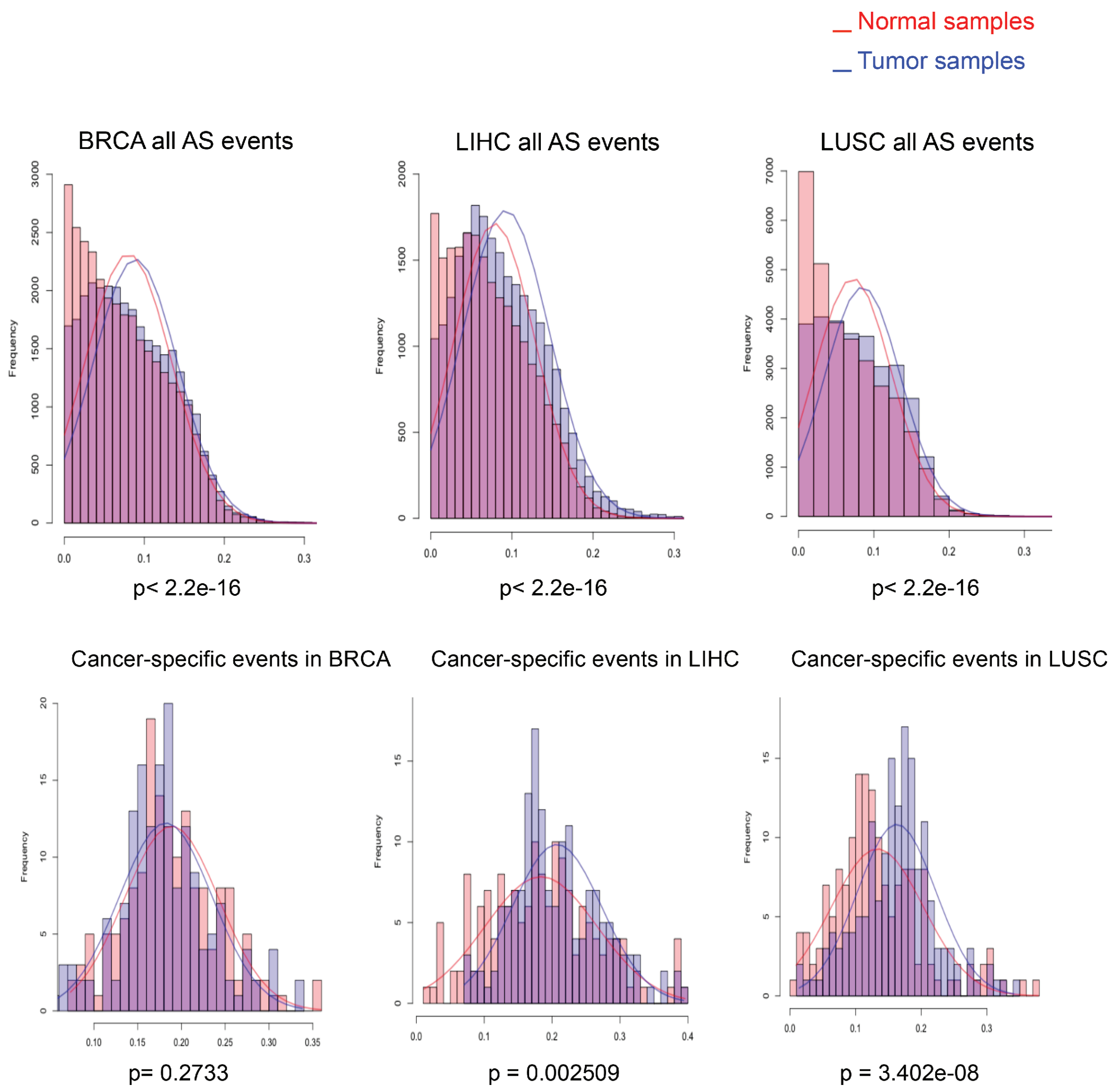

Supplementary Figure S5: Histograms of the standard deviation of PSI for all AS events (top) or for 163 cancer-specific AS events (bottom). The normal and tumor samples (in BRCA, LIHC and LUSC cancer) are plotted in different colors, and we found that for both types of AS events, the SD of PSI is larger (right-skewed) in tumor samples, suggesting that splicing in tumors are more dispersed. See also Fig 3E.

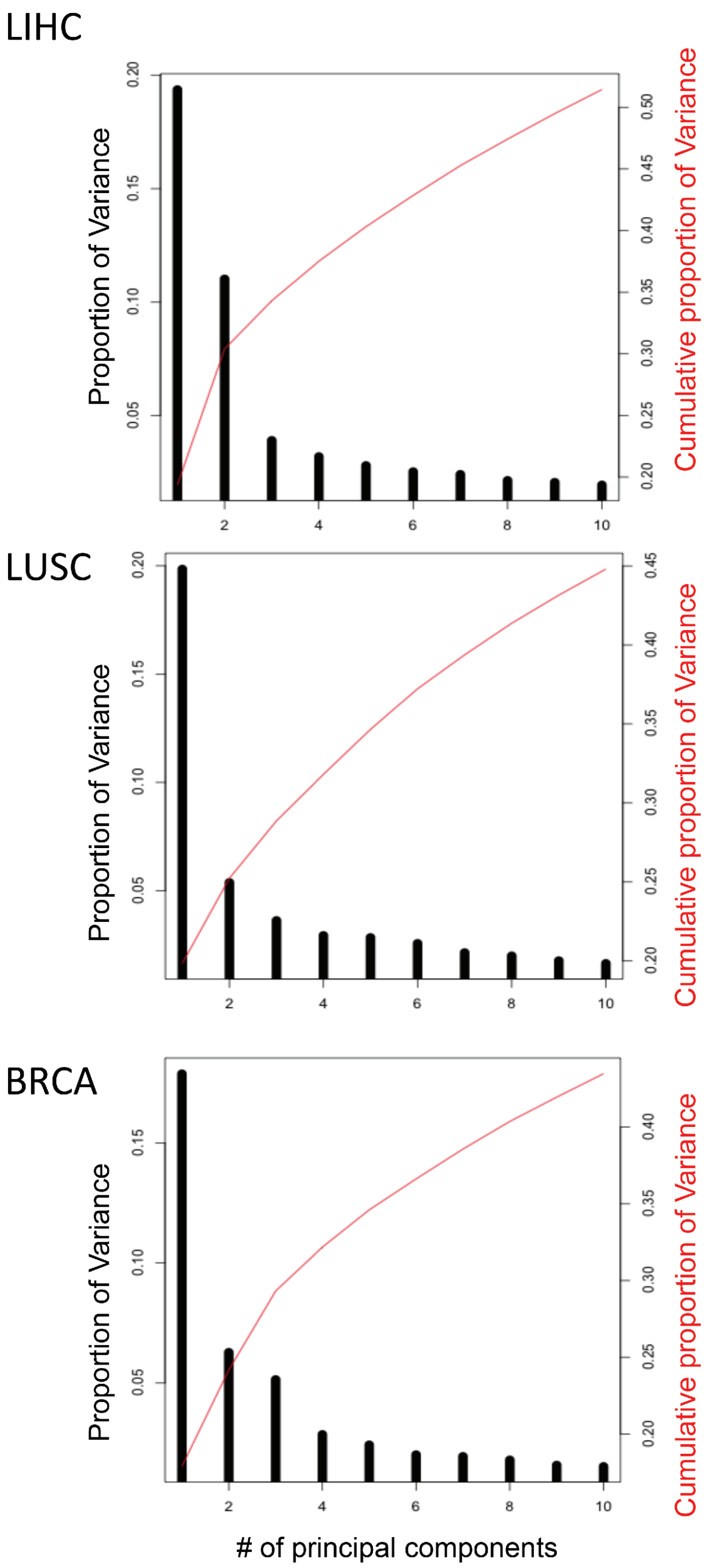

Supplementary Figure S6: The proportion of variance explained by the first ten principal components. The cumulative proportions of variance are shown in red lines.

Supplementary Table S1: 163 Cancer-specific AS events

Supplementary Table S2: MCODE Cluster results of corresponding proteins of cancer-specific AS

Cluster	Score (Density*#Nodes)	Nodes	Edges	Node IDs
1	9.28	26	116	POLE2, MYLK, LMNB1, FN1, CDCA8, ORC6L, RAD51AP1, SPAG5, PRC1, UBE2C, TUBA1A, CDKN2A, ESPL1, OIP5, AURKB, PKMYT1, TRIM59, MPHOSPH9, BIRC5, RAD54B, ATAD2, RECQL4, CDCA5, INCENP, RELN, KIFC1
2	4	14	26	FN1, SELP, ICAM3, KRT8, SPP1, COL5A1, COL6A3, CD44, NCAM1, CDKN2A, SLC3A2, RELN, ANXA2, ITGA6
3	4	10	18	HMGA1, PPARG, INSR, FN1, PTPN1, PPRC1, THRB, NCOR2, IRS1, ANXA2

Parameters:
Network Scoring:
Include Loops: false Degree Cutoff: 2
Cluster Finding:
Node Score Cutoff: 0.2 Haircut: false Fluff: true Fluff Density Cutoff 0.1 K-Core: 2 Max. Depth from Seed: 100.

Supplementary Table S3: MCODE Cluster results of proteins that are highly correlated with the cancer-specific AS

Cluster	Score (Density*#Nodes)	Nodes	Edges	Node IDs
1	32	32	496	CENPO, SPC25, NDC80, AURKB, CENPH, MLF1IP, ERCC6L, CENPE, SPC24, BUB1B, ZWILCH, ZWINT, CENPK, CENPL, KIF18A, SGOL2, BIRC5, NUF2, BUB1, CENPA, SGOL1, SKA1, INCENP, CDCA8, KIF2C, CDC20, CENPN, CASC5, CENPI, CENPM, PLK1, CENPF
2	13.53	18	115	MCM6, CLSPN, CDT1, POLE2, POLA2, MCM10, CDC6, PRIM2, MCM4, DBF4, MCM7, CDC45, ORC1L, CDC7, ORC6L, CHEK1, CCNB1, MCM2
3	5	5	10	CCNE1, TYMS, E2F1, PCNA, RRM2
4	5	5	10	UBE2T, FANCA, FANCB, FANCD2, FANCI
5	4	4	6	KIF4A, SKA3, AURKA, PRC1
6	4	4	6	NCAPG, NCAPH, NCAPD2, SMC4
7	3	3	3	HJURP, OIP5, RUVBL1
8	3	3	3	UBE2C, PSMD2, PTTG1
9	3	3	3	RFC4, TIMELESS, RFC2
10	3	7	9	MND1, CDK1, RAD51, PKMYT1, CDC25A, CDK4, CCNE2
11	3	3	3	POLR2H, SNRPA1, HNRNPL

Parameters:
Network Scoring:
Include Loops: false Degree Cutoff: 2
Cluster Finding:
Node Score Cutoff: 0.2 Haircut: true Fluff: false K-Core: 2 Max. Depth from Seed: 100.
